# Machine learning base models to predict the punching shear capacity of posttensioned UHPC flat slabs

**DOI:** 10.1038/s41598-024-54358-5

**Published:** 2024-02-17

**Authors:** Dina M. Ors, Mohamed Ramadan, Ahmed M. Farghal Maree, Amr H. Zaher, Ahmed Afifi, Ahmed M. Ebid

**Affiliations:** 1 Faculty of Engineering and Technology, Future University in Egypt (FUE), New Cairo, Egypt; 2 Faculty of Engineering, 6Th of October University, Giza, Egypt; 3 Faculty of Engineering, Ain Shams University, Cairo, Egypt

**Keywords:** Punching shear capacity, Ultra high-performance concrete, Post tensioned flat slab, Design code provisions, Artificial intelligence, Engineering, Civil engineering

## Abstract

The aim of this research is to present correction factors for the punching shear formulas of ACI-318 and EC2 design codes to adopt the punching capacity of post tensioned ultra-high-performance concrete (PT-UHPC) flat slabs. To achieve that goal, the results of previously tested PT-UHPC flat slabs were used to validate the developed finite element method (FEM) model in terms of punching shear capacity. Then, a parametric study was conducted using the validated FEM to generate two databases, each database included concrete compressive strength, strands layout, shear reinforcement capacity and the aspect ratio of the column besides the correction factor (the ratio between the FEM punching capacity and the design code punching capacity). The first considered design code in the first database was ACI-318 and in the second database was EC2. Finally, there different “Machine Learning” (ML) techniques manly “Genetic programming” (GP), “Artificial Neural Network” (ANN) and “Evolutionary Polynomial Regression” (EPR) were applied on the two generated databases to predict the correction factors as functions of the considered parameters. The results of the study indicated that all the developed (ML) models showed almost the same level of accuracy in terms of the punching ultimate load (about 96%) and the ACI-318 correction factor depends mainly on the concrete compressive strength and aspect ratio of the column, while the EC2 correction factor depends mainly on the concrete compressive strength and the shear reinforcement capacity.

## Introduction

Punching shear behaviour of the Column-slab connection using the normal reinforced concrete is usually sudden brittle failure due to the stress concentration at the small area surrounding the column. Thus, continuous research effort is performed to validate the current design recommendations available by different international design codes to make advantage of the advanced construction materials and the construction methods in such critical failure criterion^1–3^. The most common methods to avoid the brittle failure in the column-slab connections are to increase the slab or/and column dimensions using various strategies including the column head and/or drop/up panel which is not always effective especially in large spans or heavy loads in industrial buildings, bridges and even raft foundations. The advanced construction materials played an important role in improving the punching shear behavior of the column-slab connection in terms of ultimate strength, delayed cracking, and improved post-peak load energy dissipation ability. The main obstacle to using these promising materials is the design codes restricted the design equations to a limit of 69 MPa as in the EC2^4^. Thus, research work is still needed to set up design recommendations approved by different codes to validate using the advanced materials with different construction methods.

Subramanian^5^, recommended based on an extensive experimental punching shear tests of flat slabs with high strength concrete to consider the effect of the shear stud reinforcement in the Indian, ACI and the Australian design code. The author proved the important role of the shear stud reinforcement in the enhancement of the punching of the high strength concrete in terms of strength and ductile failure.

Harries^6^ experimentally tested a set of flat slabs with ultra-high concrete compressive strength and compared the results with the available ACI318 design equation. The researchers modified the ACI equation to consider the size effect, thus more accurate prediction of the ultimate punching shear strength when compared to the original ACI318. Also, the authors found that according to the fibers in the UHPC mix tend to align in the flow direction result in different flexural capacities in different directions. The developed equation is limited to the UHPC flat slabs with no axial force as in prestressed concrete or the developed axial tension force due to temperature change.

Joh et al.^7^ conducted a series of punching shear experimental tests of UHPC flat slabs restrained along the four sides. The ACI assume that the failure angle of the major punching crack is 45°, while the researchers found that it could be reduced to be 38° in case of prestressing the flat slabs. The equations using this assumption predict more accurate ultimate load when compared to the conventional design equations in the ACI. Also, the authors found that more research is needed to study the difference between conventional concrete and the UHPC, especially the post cracking behavior when flexural failure is expected.

Metwaly^8^ examined the validity of the ECP-203, ACI-318, and BS-8110 codes provisions on the punching shear capacity of fifty-five flat slabs. The authors found that the parameters of slab thickness, tension reinforcement ratio and concrete compressive strength have significant effect of the punching shear behavior in terms of ultimate load and maximum deflection. Also, the British Code (BS-8110) formula gives more accurate prediction of the punching shear capacity for both NSC and HSC flat slabs than ACI-318 and ECP-203 codes.

Elsanadedy et al.^9^ proposed an innovative design equation to predict the punching shear strength of flat slabs with high strength concrete (HSC). The developed equation is limited to HSC flat slabs with depth not more than 300 mm and concrete compressive strength below 120 MPa. Authors recommended considering the size effect as the ratio between the effective depth to the critical perimeter (*d/bo*) and the reinforcement ratio in design codes such as the ACI.

Inácio et al.^10^ compared the results of the experimentally tested flat slabs with concrete compressive strength ranged from 36 to 130 MPa with different design codes provisions. The ACI and the EC estimate ultimate punching strength higher than the experimental results with smaller failure perimeter. However, MC2010 is always conservative.

Ricker et al.^11^ introduced a new proposal based on the current design codes design equations to differentiate between the punching shear failure mechanisms that occur inside and outside the interior column-slab connection strengthened with UHPC column cap. Authors conducted a set of experiments to investigate the effect of the strengthening at column-slab connection and compared the test results with the available FIP model and the EC design equations. The researchers recommended a proportional factor of 1.77 and 2.64 to be multiplied by the ultimate punching strength calculated by the current design equations provided by the FIB and the EC respectively.

Zhi et al.^12^ experimentally tested nine of UHPC flat slabs that failed due to punching shear. The main variables between the tested UHPC flat slabs are slab thickness, concrete strength, column-slab contact area, reinforcement ratio and loading position to consider the load eccentricity. Feasibility of different codes in prediction of punching shear need to be updated to be applicable in case of UHPC flat slabs. Based on the experimental results, the shear span-to-depth ratio highly affected prediction of the two-way UHPC punching shear, thus the authors proposed design equation which considered the effect of shear span-to-depth ratio which highly affected the punching shear failure in the UHPC flat slabs.

Qi et al.^13^ investigated the effect of using partial and full depth column cap in the column-slab connection using the superior advantages of the UHPC. Authors experimentally tested a series of interior column-slab connection strengthened with variable depth of the UHPC column cap. The test results were compared to the current design code equations including the ACI and FIP model design equations. As expected, the design codes underestimated the ultimate punching shear strength by 0.44 and 0.67 respectively. The researchers developed an analytical model based on yield line theory is proposed to estimate the punching shear strength of the flat slabs.

Abdulqader et al.^14^, proposed design equation to predict the punching shear strength of reinforced concrete flat slabs with compressive strength of 14.4–119 MPa that is out of the available range by the current design codes. The concrete compressive strength of the reinforced concrete flat slabs included in this study ranged from MPa to MPa which exceeded the limits of the ACI and the Australian Concrete Structures Standard (AS-94) of 69 MPa and 50 MPa respectively. The authors concluded that the proposed model is highly reliable, and the safety of different design codes sharply dropped when comparing the predicted punching shear strength by the design codes to the experimentally tested flat slabs.

Ebid et al.^15^ predicted the punching shear strength of lightweight concrete flat slabs using means of artificial intelligence including artificial neural network (ANN), genetic programming (GP), and evolutionary polynomial regression (EPR). The developed design expression showed the complicated inter-relation between affective variables namely, namely, the flat slab thickness, column dimensions, concrete density, concrete strength and the grade and ratio of the steel reinforcement.

Yehia et al.^16^ carried out a series of punching shear tests of UHPC flat slabs. The concrete compressive strength ranged from 164 to 193 MPa. These high concrete grades were achieved by adding steel microfibers that led to an increase of concrete strength from 70 MPa to two and half this value. Steel fibers enhanced the ductility of the flat slabs and reduced the failure angle of the punching shear cone. Both design codes of ACI and the EC predicted ultimate load larger than the experimentally monitored. Thus, the current available equations need to be modified. The authors validated a finite element model against the experimental results with maximum difference of + 2.5% in ultimate shear strength.

Ramadan et al.^2^ experimentally tested the punching shear behavior of the post-tensioned UHPC flat slabs to investigate the influence of various parameters including the concrete compressive strength and the strands lay out in both directions. The bundle & gapped-bundle layout slab showed a slight enhancement in the ultimate punching capacity due to improving the efficiency of used strands, but also showed major reduction in deflection, angle of shear crack and dissipated energy. The researchers compared the test results with the design equations of the ACI and the EC that lead to the (HSC) slabs showing good agreement with both ACI & EC2, while (UHPC) slab exceeded the theoretical capacity by 65%.

Afifi et al.^3^ compared the results of the experimentally testes post-tensioned UHPC flat slabs with the predicted ultimate punching shear using the available equations in ACI and EC2 that showed the significant difference between the predicted and the experimental results. The ACI.

predictions are more accurate while EC2 predictions are more conservative. The average deviation was about 1% and 6% for ACI and 23% and 14% for EC2 for slabs without and with punching reinforcement respectively.

El Zareef et al.^17^ investigated the punching shear behavior of ultra-high performance self-compacting concrete slabs UHPSC flat slabs by experimentally testing a series of flat slabs varying in concrete type, slab thickness, and reinforcement ratio on the punching shear capacity. All current codes lead to conservative results when compared with the experimental ultimate shear strength of UHPSCC flat slabs.

Yeh^18^ used the Artificial Neural Network (ANN) to investigate the effect of concrete mix proportions to achieve the required concrete compressive strength to develop ultra high-performance concrete. Authors proved that ANN are reliable and easier to the conventional experimental and numerical investigation based on the regression analysis.

Nikoo et al.^19^ conducted analytical study to investigate the reliability of using the artificial intelligence as a combination of ANN and Genetic Algorithm (GA) to predict concrete compressive strength under the effect of different parameters including coarse and fine gravel maximum size, W/C ratio, amount of gravel and coefficient of soft sand parameters. The authors validated the developed model against a set of experimental and numerical models using multiple Linear Regression (MLR) and proved that the developed model is more accurate and flexible in predicting concrete compressive strength.

Pishro et al.^20^ applied an Artificial Neural Network (ANN) to precisely expect the bond stress between the reinforcing steel bars and the surrounding UHPC. The authors developed a design equation to calculate the local bond stress based on an experimental test and developed finite element model using ABAQUS. Main variables were concrete compressive strength, bond length, concrete cover and the rebar diameter. The ANN algorithm proved that the predicted local stress equation is accurate and can be used in design.

Kim et al.^21^ used Smooth Particle Hydrodynamics to predict the amount of fragment and travel distance of concrete barrier under impact loading. Then the researchers developed an ANN and Multi Linear Regression models to investigate the accuracy of the conducted study. Results showed that the ANN model achieved better coefficient of determination than the MLRM. Finally, the researchers developed the concrete fragility curves not to exceed the specific amount of fragment and travel distance of concrete barrier under the same value of the impact loading.

Bakhoum et al.^22^ validated an ANN model to predict the concrete compressive strength achieving sustainable criteria including compressive strength, carbon dioxide and cost. As the proposed concrete contains proportions of cement kiln dust and fly ash. The input parameters are based on more than hundred-fifty concrete mixes collected from previous studies. Then, TOPSIS strategy has been used to predict the best mixture proportions to produce a green sustainable concrete mix.

The previous review showed many studied concerned in punching in PT-slabs, and UHPC-slabs, but it indicated a knowledge gap regarding the punching behavior of PT-UHPC flat slabs. Hence, the main objective of this research is to extend the applicable range of the current design codes provisions regarding punching shear capacity of flat slabs to include the PT-UHPC. The considered design cods are (ACI-318 & EC2). To achieve this goal, two correction factors (one for each code) were developed using combined (FEM-AI) technique^23,24^

## Methodology

To achieve the research goal, a three phases methodology were used as follows:

Phase-1 was concerned in developing a typical FEM model for PT-UHPC flat slab considering concrete compressive strength (Fcu), strands layout (in terms of the average pre-stressing stress in the punching zone) (Fps), shear reinforcement capacity (Ash.Fy) and the aspect ratio of the column (L/B). Then the developed model was validated using the experimental results from the previous research work^2,3^.

In Phase-2, a full parametric study (includes all parameters combinations) was conducted to generate two databases (one database for each considered design code). Each database includes the considered parameters besides the correction factor (the ratio between the design code punching capacity and the FEM punching capacity).

**Figure 1:**
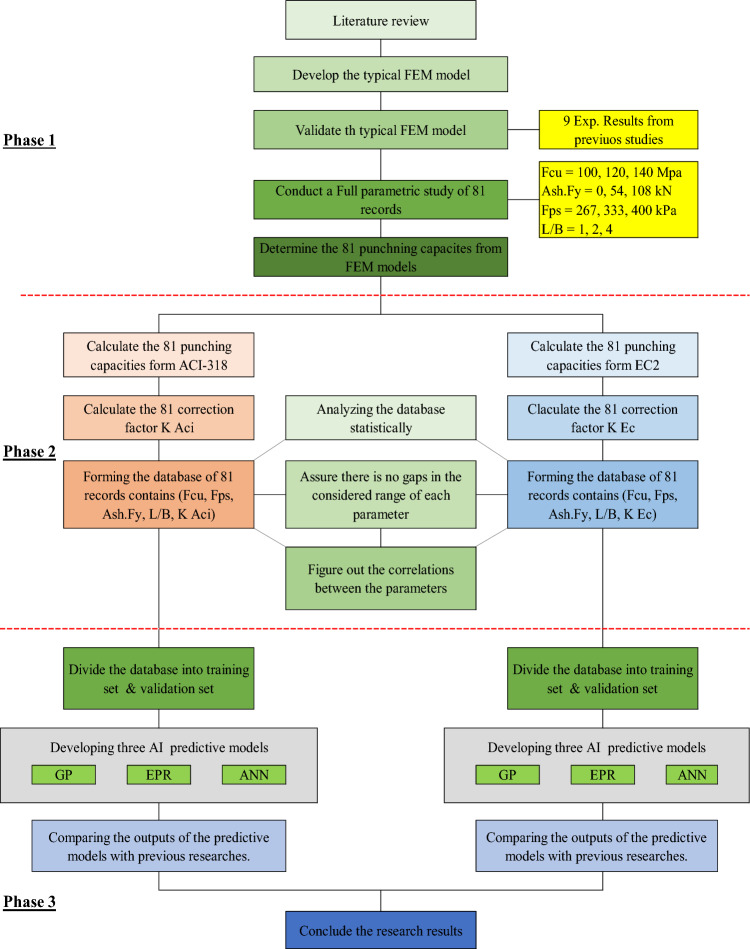
The applied methodology.

Finally, in Phase-3, three AI techniques namely “Genetic programming” (GP), “Artificial Neural Network” (ANN) and “Evolutionary Polynomial Regression” (EPR) were applied on each database to predict the correction factor in terms of the considered parameters. Figure 1 graphically presents the used methodology.

## Phase 1: Develop and validate the Typical FEM model 

### Collecting experimental test results for validation

#### Flat slabs specimens’ description

**Figure 2:**
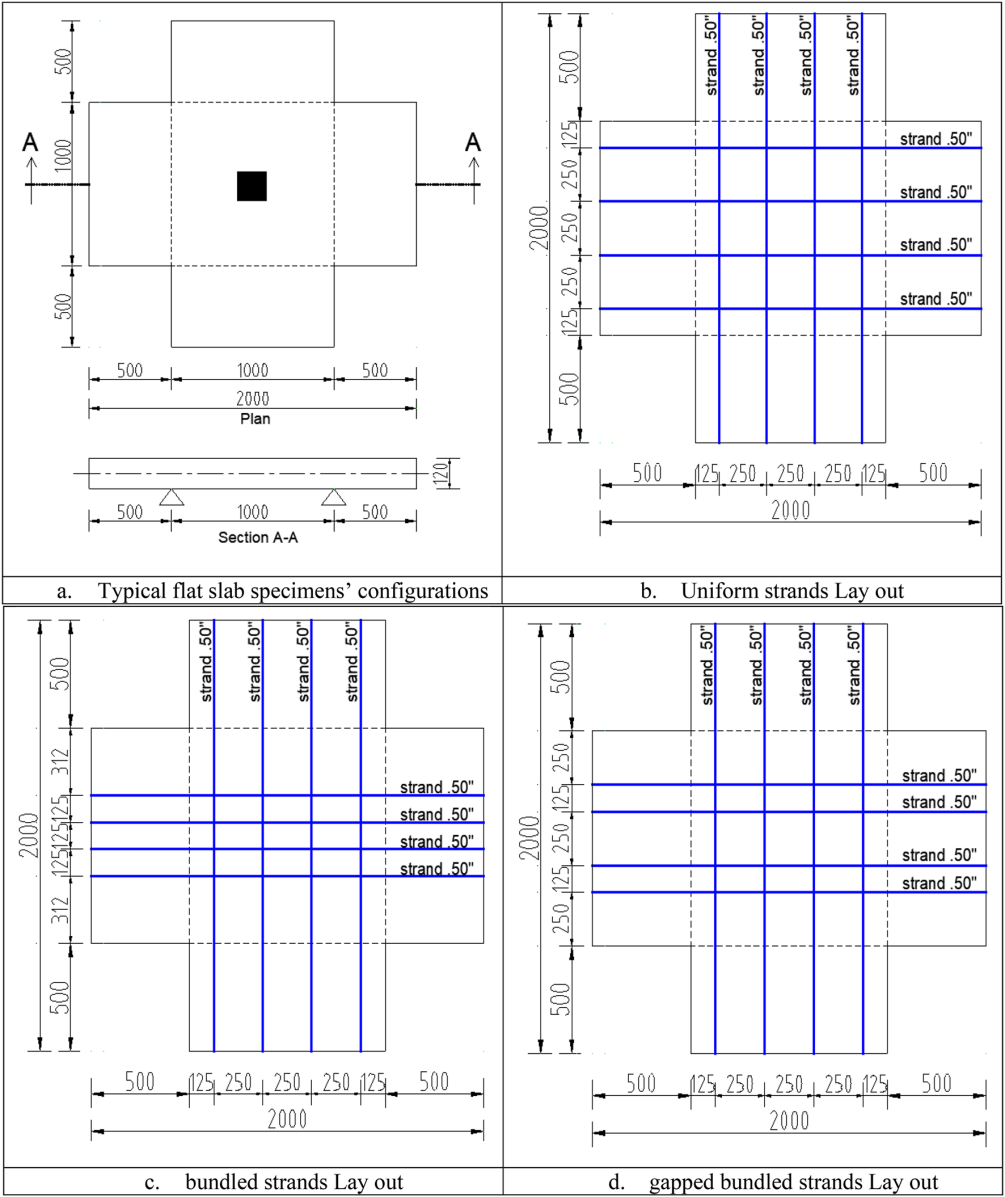
The configurations of tested flat slabs (all dimensions are in mm).

The developed typical FEM model will be validated against the experimental results of monotonic testing of nine PT-UHPC flat slabs from previous studies by the same research group^2,3^. The dimensions of all tested slabs were 1000 mm × 1000 mm in plan and thickness of 120 mm, they all had four 0.5″ posttensioning strands in each direction stressed up to net effective stress of 800 MPa after losses. All slabs were supported along their edges and subjected to monotonic vertical load at their midpoints through loading steel block till failure. The main variables between the tested slabs are the concrete compressive strength, column dimensions aspect ratio, vertical reinforcement ratio and the strands lay out. Details of all specimens are summarized in Table 1. The configurations of tested flat slabs are illustrated in Fig. 2.

**Table 1:** The configurations of tested flat slabs^2,3^.

Slab	fcu	Column Dim	L/B	Punching reinforcement (RFT)	Ash.Fy	Strands	fps
ID	(MPa)	(mm x mm)	(−)		(kN)	Layout	(kPa)
S1	**120**	50 × 50	1	–	0	Uniform	267
S2	**100**	50 × 50	1	–	0	Uniform	267
S3	**140**	50 × 50	1	–	0	Uniform	267
S4	120	50 × 50	1	–	0	**Gapped**	**333**
S5	120	50 × 50	1	–	0	**Bundled**	**400**
S6	120	50 × 50	1	**2ɸ6@75 mm**	**108**	Uniform	267
S7	120	50 × 50	1	**2ɸ6@150 mm**	**54**	Uniform	267
S8	120	**50 × 200**	**4**	–	0	Uniform	267
S9	120	**50 × 100**	**2**	–	0	Uniform	267

#### Materials

**Concrete:** The concrete mixes of the tested UHPC flat slabs included concrete compressive strength of 100 MPa, 120 MPa and 140 MPa. Each mix was tested under compression and tension in the same test day to ensure slabs strength. Tables 2 and 3 summarize the mixes proportions and mechanical properties of all concrete mixes.

**Table 2:** Mix proportion of Ultra-High-Performance Concrete.

Mix	Cement CEM 52.5 N	Silica fume	$\frac{{\mathrm{sand}}}{\mathrm{total aggregate}}$	$\frac{\mathrm{Quartz powder}}{\mathrm{total aggregate}}$	$\frac{\mathrm{coarse aggregate }}{\mathrm{total aggregate}}$	W/C	Add superplasticizer	Steel fiber	Curing
	kg/m^3^	%	Siliceous		Dolomite–D5*		%	%	
**100**	800	20	0.25	0.25	0.5	0.16	4	1	water
**120**	800	20	0.25	0.25	0.5	0.16	4	1	SC-7d ˟
**140**	800	20	0.25	0.25	0.5	0.16	4	3	SC-7d ˟

*D5: maximum diameter equals 5 mm.

˟Sc-7d: Steam curing for 7 days.

**Table 3:** Mechanical properties of UHPC concrete mixture.

	Average of compressive Strength (*f*_*c*_ ) (MPa)	Average of the indirect tensile strength (*f*_*tm*_) (MPa)
Mix 100	98.5 MPa	8.25
Mix 120	119 MPa	10.50
Mix 140	138	16.50

**Steel:** The experimentally tested flat slabs were reinforced by the posttensioned strands without any main mild reinforcement. The installed strands were low relaxation 7-wire strands with 0.5″ diameter. The required accessories were complaint with the ASTM A416 "Standard Specification for Steel Strand, Uncoated Seven Wire Strand for Prestressed Concrete"^24^.

### Developing the typical FEM model

#### Modelling strategy

The finite element model (FEM) performed in this study is developed using the available package of ABAQUS software. The main approach of the developed FEM in this study is to catch the experimentally tested slabs to verify the applied model parameters, then set up the required parametric study using wider range of the experimentally tested variables. The developed FEM followed the conventional modelling methodology of simulating concrete structure as 3D solid elements and the steel reinforcement (strands) as embedded region in the surrounding concrete (Host region). Loading area is defined on the top surface of the concrete slab according to the required column dimensions. The model runs till the analysis is aborted according to the input plastic parameters indicating slab full failure. The details of the developed finite element model are all discussed herein this section.

#### Geometry and meshing sensitivity analysis

**Figure 3:**
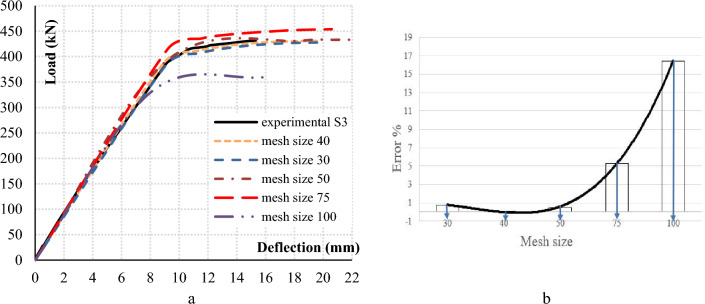
Comparison between the experimental and the FEM with different mesh size.

The geometry of the developed finite element model is defined in cartesian coordinate system by X–Y plane and Y-axis pointing upward in the model height direction. Elements required to simulate the concrete flat slabs must deform freely till failure without any shear locking restrictions that arises in first order fully integrated elements, thus 3D solid elements quadratic hexahedral brick 20-noded with reduced integration (C3D20R) are chosen to model concrete elements. Post-tensioned strands carrying high levels of tensile stresses are modeled using three-dimensional two node first order truss element to simulate the axial deformability and stiffness. Considering the full bond between the embedded strands and the surrounding concrete elements, mesh size of both concrete and steel strands must be the same. Mesh size used in this study was chosen based on a sensitivity analysis conducted to decide the best and most accurate response. Flat slab S3 was modeled using different mesh sizes for both concrete and steel strands of 30 mm, 40 mm, 50 mm, 75 mm, and 100 mm. Figure 3a compares the load–deflection response of each model against the experimental results of the same flat slab S3 which clearly shows that the model with mesh size of 40 mm achieves the least error when compared to the experimental response. Also, the punching shear strength of the tested flat slab is the closest to the model with mesh size of 40 mm as shown in Fig. 3b.

#### Concrete material model

In compression, concrete is linear elastic and defined by the concrete Young’s modulus according to experimental tests and concrete Poisson’s ratio (ν)^2,3^.

**Figure 4:**
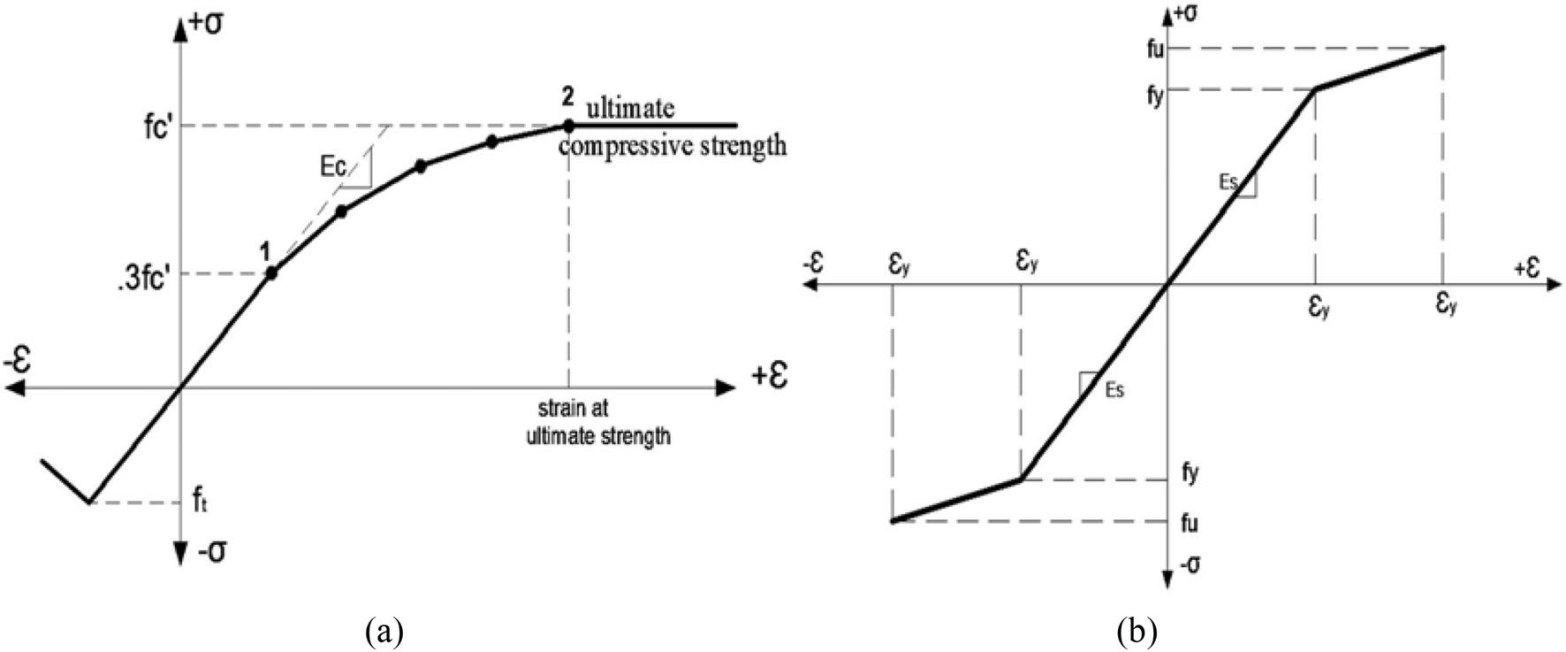
Stress–strain curve of used material.

The plastic portion is defined using the Concrete damaged plasticity CDP material model embedded in ABAQUS^25–28^. To fully define the plastic behavior in CDP, yielding criterion is defined using a set of plasticity parameters. These plasticity parameters are, the dilation angle Ψ in the p-q plane, Flow potential eccentricity, the eccentricity (ϵ) is a small positive number that defines the rate at which the hyperbolic flow potential approaches its asymptote, the ratio of initial equi-biaxial compressive yield stress to initial uniaxial compressive yield stress (f_b0_/f_c0_), the ratio of the second stress invariant on the tensile meridian to that on the compressive meridian, the flow potential eccentricity, the ratio of initial biaxial compressive yield stress to initial uniaxial compressive yield stress, the ratio of the second stress invariant on the tensile meridian to that on the compressive meridian and the viscosity parameter that defines viscoelastic regularization and the viscosity parameter μ used for the visco-plastic regularization of the concrete constitutive equations in Abaqus/Standard analyses^25^. Then, concrete behavior is defined in compression and tension. The concrete compressive behavior is defined using Eqs. 1, 2 and 3 that are validated by Choi et al.^29^ In this model, the plastic behavior starts at 0.3 f_c_^’^ and the stress–strain curve is nonlinear as shown in Fig. 4a. Point 1 is defined in the elastic range as 0.3 f_c_′ and dividing this value the concrete young’s modulus (E_c_) to get the strain at the end of the elastic zone as in Eq. 1. Points from point 1 to point 2 are obtained using Eq. 2, then Eq. 3 is used to calculate the ultimate strain at which the curve ends. The tensile behavior of concrete is defined as linear elastic using Eq. 1 till the concrete tensile strength then linear decrease of concrete tensile strength till zero at the ultimate tensile strain. Concrete tensile strength is experimentally determined^2,3^ as well as all the input parameters of different concrete grades 100 MPa, 120 MPa and 140 MPa are summarized in Table 4.

1 $$ {\mathrm{E}}_{{\mathrm{c}}} = \frac{{\mathrm{f}}}{\xi } $$

2 $$ {\mathrm{f}} = \frac{{{\mathrm{E}}_{{\mathrm{c}}} \xi }}{{1 + \left( {\frac{\xi }{{\xi_{0} }}} \right)^{2} }} $$

3 $$ \xi_{0} = \frac{{2{\mathrm{f}}_{c}^{\prime } }}{{{\mathrm{E}}_{{\mathrm{c}}} }} $$

where, f: stress at any point of strain ξ (Psi), ξ: strain at any stress f, ξ_0_: strain at the ultimate compressive strength ${f}_{c}{\prime}$.

**Table 4:** Parameters of the Concrete material model (CDP) according to the concrete grade

Concrete compressive strength		Elastic parameters		Plastic parameters				Tensile strength
$${f}_{cu}^{*}$$ (MPa)	$${f}_{c}^{{\prime}**}$$(MPa)	$${E}_{c}$$(MPa)	$${\nu }_{c}$$	Ψ	ϵ	f_b0_/f_c0_	μ	$${f}_{t}$$(MPa)
100	80	40,600	0.20	50.50	1.16	0.667	0.001	8.25
120	96	40,600						10.5
140	112	45,300						16.5

*cube compressive strength (MPa).

**cylinder concrete compressive strength (MPa) equivalent to 80% of f_cu_.

#### Steel material model

Steel reinforcement is modeled using 2D-truss element (T3D2) to simulate the axial stiffness and deformations. The mesh size of all steel elements is 40 mm based on a sensitivity analysis that best fits the experimental results, also the steel has the same mesh size as the surrounding bonded concrete elements. The steel material model is defined as elastic–plastic with strain hardening material for both mild reinforcement (RFT) in stirrups and the High Tensile Steel (HTS) in strands^30^. The steel Elastic parameters are Young’s modulus of 20 GPa and 200 GPa for the mild and HTS in case of stirrups and strands respectively and the Poisson’s ratio is 0.30 in both steel grades. The yield strength of the mild and HTS are 240 MPa and 1600 MPa with ultimate strength of 360 MPa and 1800 MPa respectively. Strands and the stirrups are embedded and fully bonded with the surrounding concrete. Figure 4b shows the steel material model used in the FEM.

#### Loading and boundary conditions

**Figure 5:**
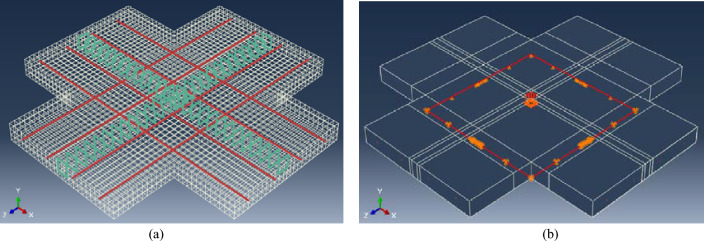
Details of the developed FEM.

Full scale models of the experimentally tested flat slabs are simply supported along its edges with line roller-hinged boundary conditions in each direction. The pre-stress is applied to the bonded strands in each direction as an initial condition, then the monotonic column load is simulated by an incremental displacement boundary condition in negative global Y-direction. Typical finite element model elements, embedded bonded strands, stirrups and applied displacement direction are shown in Fig. 5.

### Validating the typical FEM model

#### Failure Mechanism

**Figure 6:**
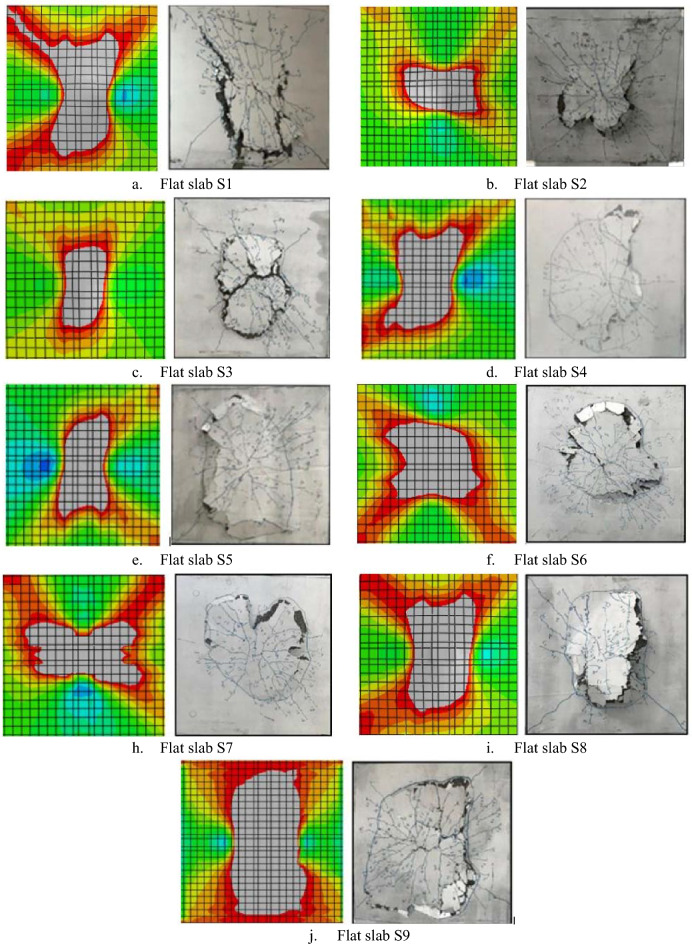
Comparison between Failure mechanism of the experimental tests and the FEM.

All tested flat slabs failed due to punching shear in both experimental and the developed finite element model. Figure 6 shows clearly that the FEM successfully followed the conventional punching failure in terms of crack propagation and the major punching cone perimeter at the bottom surface of the tested flat slabs.

#### Load–Deflection (P-∆) response

**Figure 7:**
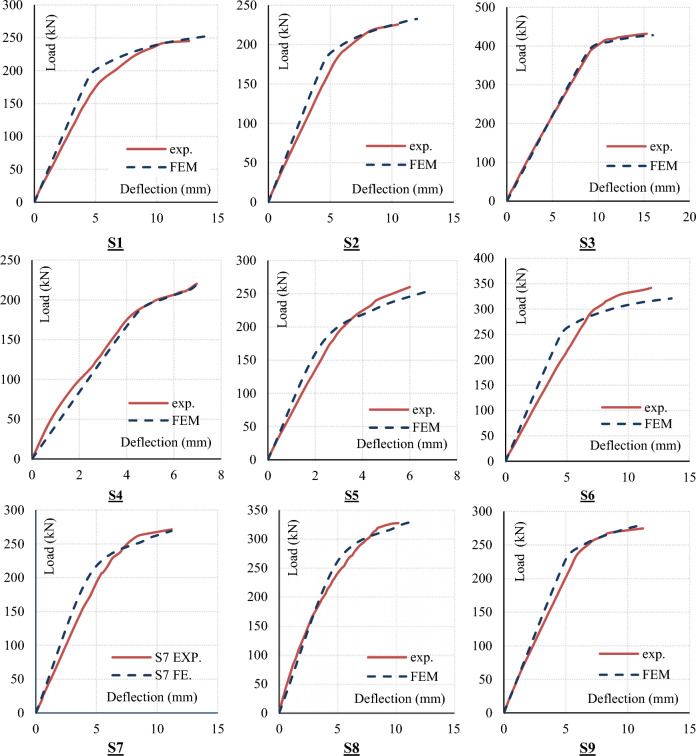
Comparison between the experimental and the FEM load–deflection curves.

Each tested slab was modelled using the typical FEM model and loaded till failure, both ultimate load and midpoint ultimate deflection were recorded for each slab besides its (Load–deflection) curve. These outputs were compared with experimentally measured values to verify the typical FEM model. Figure 7 and Table 5 summarize this comparison.

**Table 5:** Comparison between F.E & EXP. Results (Pu & Δu).

slab	Pu (kN)			Δu (mm)		
	F.E	EXP	F.E/ EXP	F.E	EXP	F.E/ EXP
S1	255.6	245.0	104.3	14.5	12.7	114.2
S2	241	226	106.6	12.05	10.5	114.8
S3	430.86	431	100.0	15.96	15.3	104.3
S4	260.54	250	104.2	11.37	7.7	147.7
S5	272.2	260	104.7	6.8	6.0	113.3
S6	320.9	341.5	94.0	13.52	11.9	113.6
S7	276.13	271.4	101.7	11.18	11.2	99.8
S8	343.86	327.4	105.0	14.28	10.2	109.5
S9	290.63	274.7	105.8	11.13	11.2	99.4
	Average		102.9	Average		113.0
	Standard deviation		3.91	Standard deviation		14.39
	Variance		3.8%	Variance		12.7%

The results showed that the difference in the outputs between experimental and FEM models are about 3.8% and 12.7% for ultimate load and deflection respectively. This very good matching indicates a high reliability of the developed FEM models in terms of ultimate punching load.

#### Stiffness and Dissipated Energy

**Figure 8:**
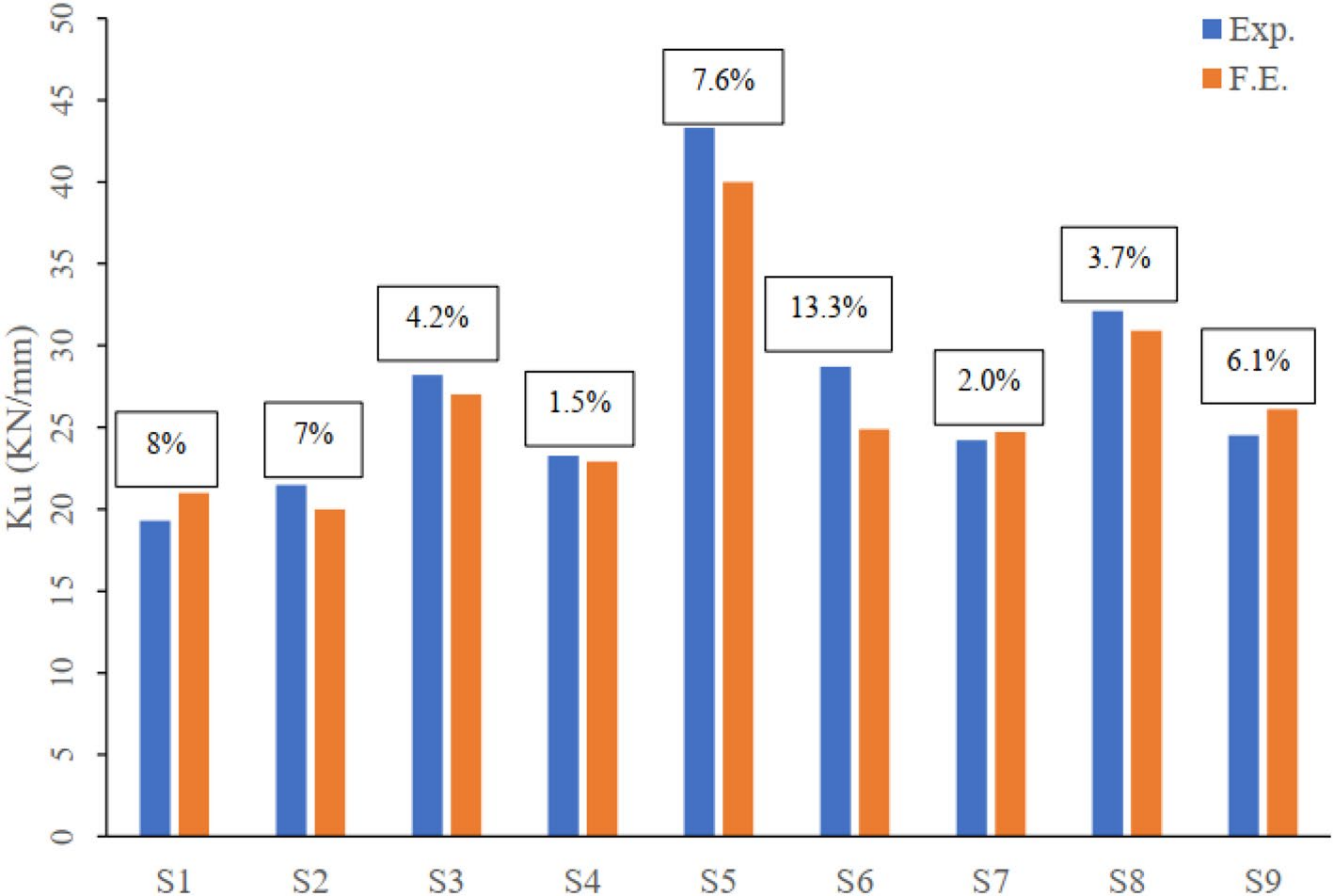
Comparison between experimental and FEM ultimate stiffness of all tested flat slabs.

**Figure 9:**
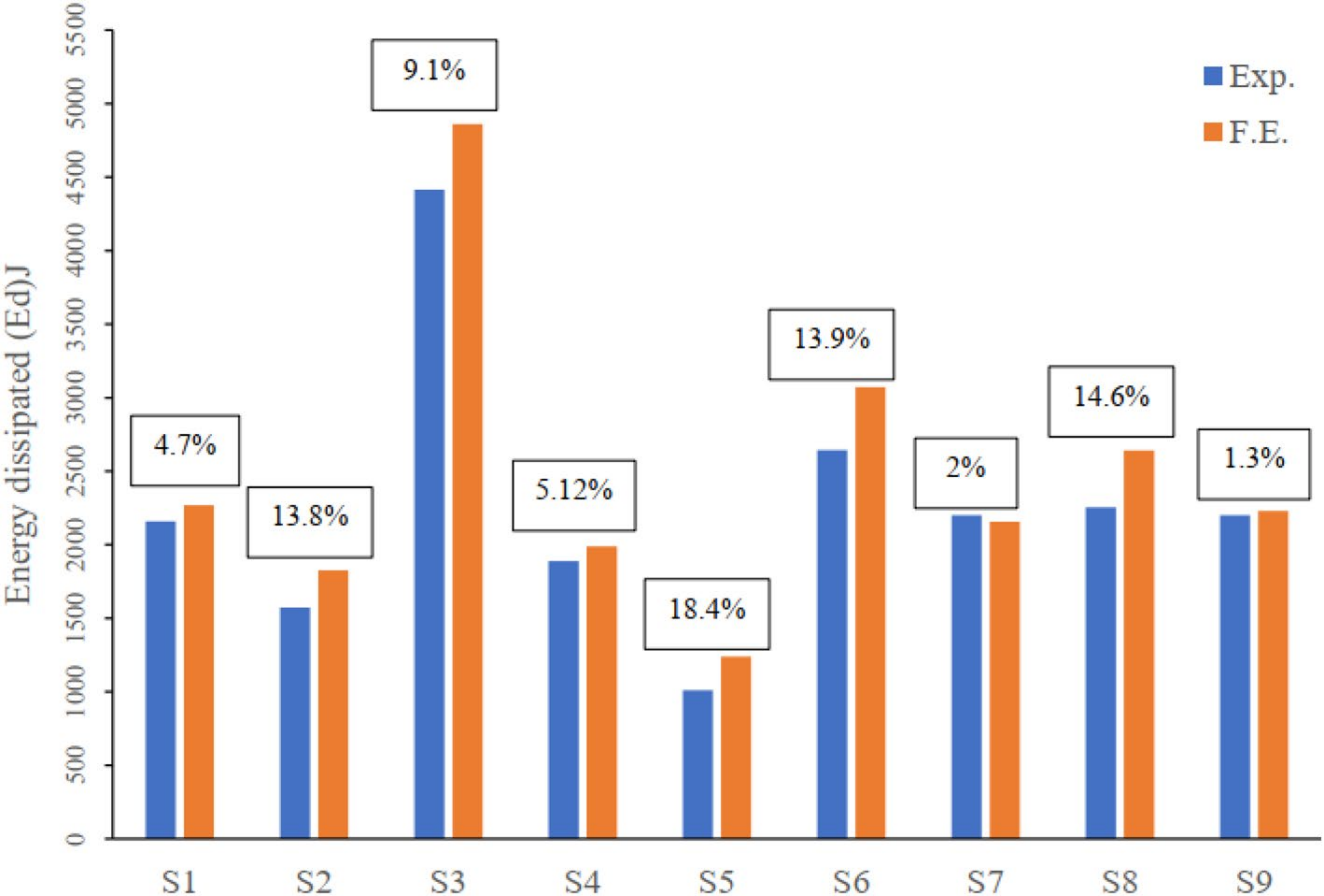
Comparison between experimental and FEM dissipated energy of all tested flat slabs.

As an extension of the finite element model validation study, the stiffness at failure and the exerted dissipated energy for each modeled flat slab have been calculated and compared to the experimental values. Stiffness is calculated as the ratio between the ultimate load and the maximum deflection at the same loading level. And the area under the load–deflection curve till point of failure represented the dissipated energy. The maximum difference between the FEM and the experimental is 13.3% and 18.4% the ultimate stiffness and the dissipated energy respectively. Figures 8 and 9 compare the values of the stiffness and the dissipated energy respectively as well as the error between the experimental and the FEM of each modeled flat slab.

## Phase 2: Parametric study

The aim of this section is to conduct a full parametric study which includes enough combinations to cover the considered ranges of the involved parameters. The selected values for each parameter were extracted from the previously published experimental work^2,3^ as follows:

- Concrete compressive strength (Fcu) = 100, 120, 140 MPa
- Average pre-stressing stress in the punching zone (Fps) = 267, 333. 400 kPa
- Shear reinforcement capacity (Ash.Fy) = 0, 54, 108 kN
- Aspect ratio of the column (L/B) = 1.0, 2.0, 4.0

**Figure 10:**
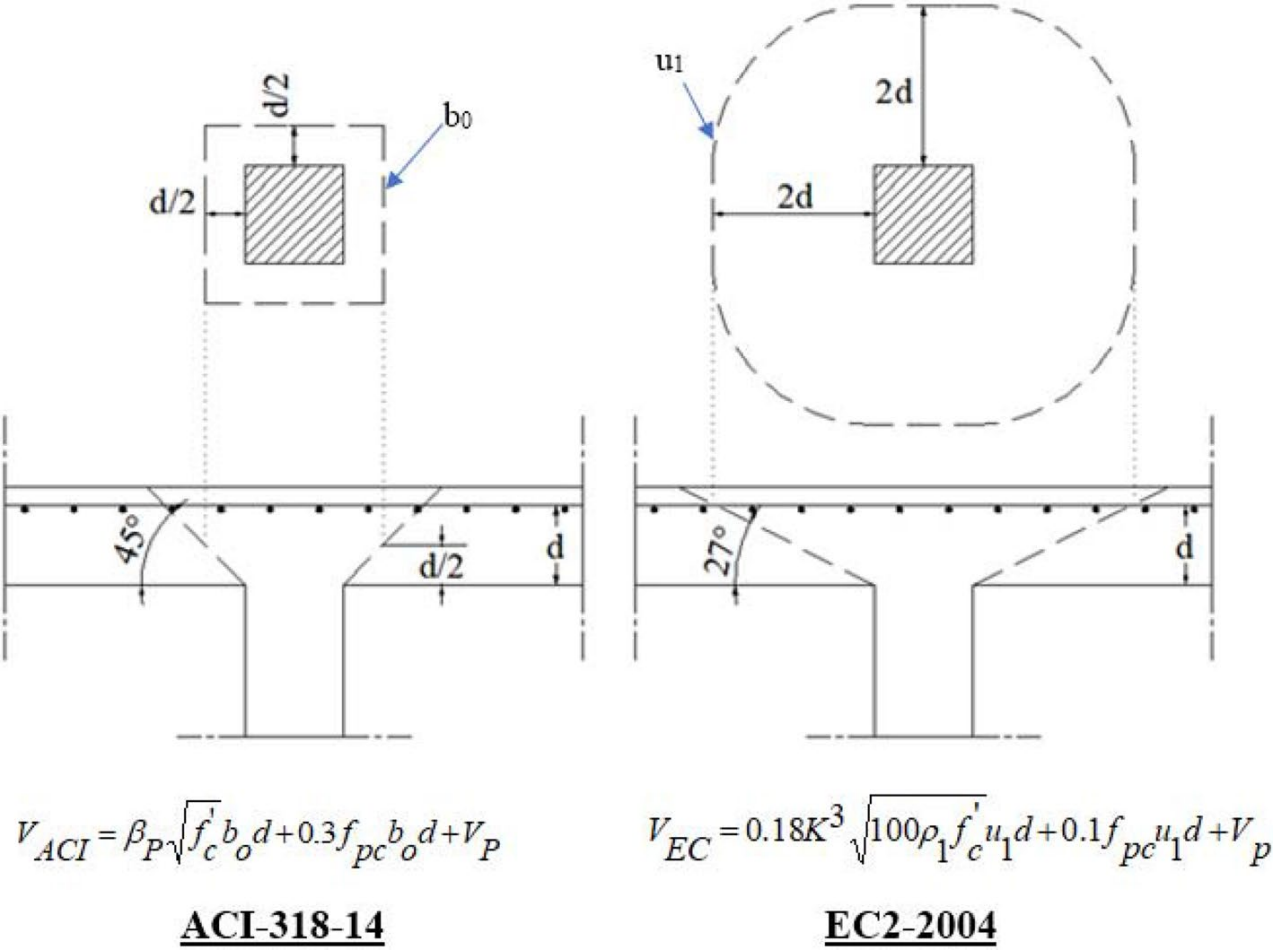
Punching capacity formulas as per ACI-318 and EC2.

Eighty-one FEM models were developed, one model for each parameters combination to determine its FEM ultimate capacity. Then, the punching capacity of each parameter’s combination were calculated using ACI-318 and EC2 as shown in Fig. 10. Finally, the correction factors (K Aci = Pu FEM / Pu Aci) and (K Ec2 = Pu FEM / Pu Ec2) were calculated for each parameter’s combination.

### The generated database 

The generated database forms the parametric study contains 81 records; each record contains the following data:

Fcu: The cube characteristic strength of concrete (MPa)

L/B: The aspect ratio of the column (length/width)

Ash.Fy: Ultimate tensile capacity of shear reinforcement within the punching zone (2d+B+2d) in (kN)

Fps: The average pre-stressing stress (in both directions) within the punching zone (2d+B+2d) in (kPa)

K Aci: The correction factor for ACI-318 formula (Predicted capacity/ACI Capacity)

K Ec2: The correction factor for EC2 formula (Predicted capacity/EC2 capacity)

**Figure 11:**
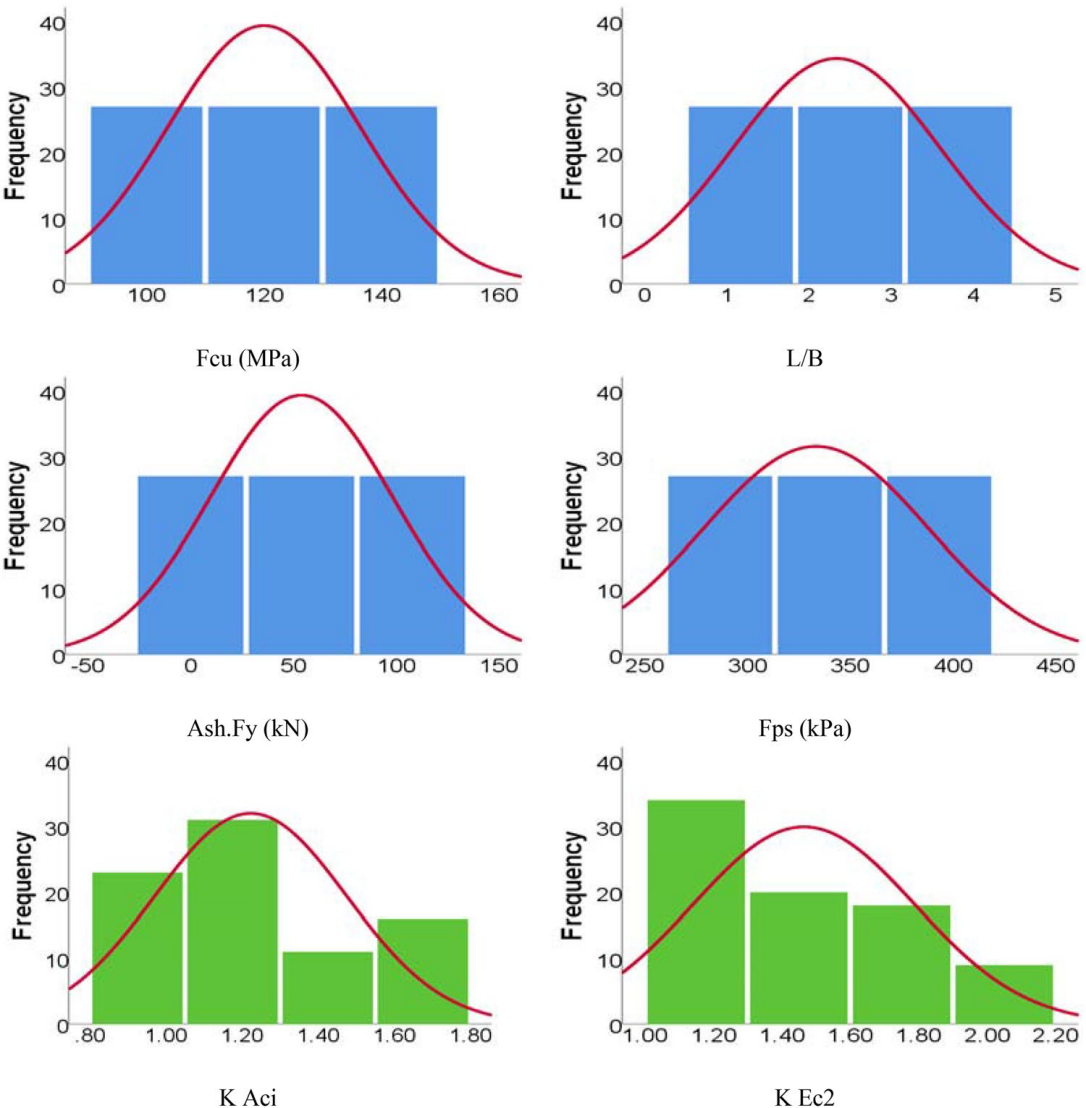
Distribution histograms for inputs (in blue) and outputs (in green).

The generated records were divided into a training set (65 records) and validation set (16 records) as recommended by Ebid ^31^. The “Appendix” includes the complete dataset, while Tables 6 and 7 summarize their statistical characteristics and the Pearson correlation matrix. Finally, Fig. 11 shows the histograms for both inputs and outputs. The complete database is attached in the “Appendix”.

**Table 6:** The statistical characteristics of the utilized database.

	Fcu (MPa)	L/B	Ash.Fy (kN)	Fps (kPa)	K Aci	K Ec2
Training set						
Min	100.00	1.00	0.00	267.00	0.95	1.09
Max	140.00	4.00	108.00	400.00	1.69	2.06
Avg	118.77	2.38	54.83	333.34	1.21	1.45
SD	16.78	1.26	42.88	54.71	0.24	0.31
VAR	0.14	0.53	0.78	0.16	0.20	0.22
Validation set						
Min	100.00	1.00	0.00	267.00	0.93	1.14
Max	140.00	4.00	108.00	400.00	1.77	2.07
Avg	125.00	2.13	50.63	333.31	1.27	1.52
SD	13.23	1.17	48.56	52.57	0.28	0.35
VAR	0.11	0.55	0.96	0.16	0.22	0.23

**Table 7:** Pearson correlation matrix of the utilized database.

	Fcu	L/B	Ash.Fy	Fps	K Aci	K Ec2
Fcu	1.00					
L/B	0.00	1.00				
Ash.Fy	0.00	0.00	1.00			
Fps	0.00	0.00	0.00	1.00		
K Aci	0.84	− 0.27	− 0.02	0.05	1.00	
K Ec2	0.86	0.12	− 0.26	0.05	0.88	1.00

## Phase 3: Applying the (AI) techniques

### (AI) Techniques 

**Figure 12:**
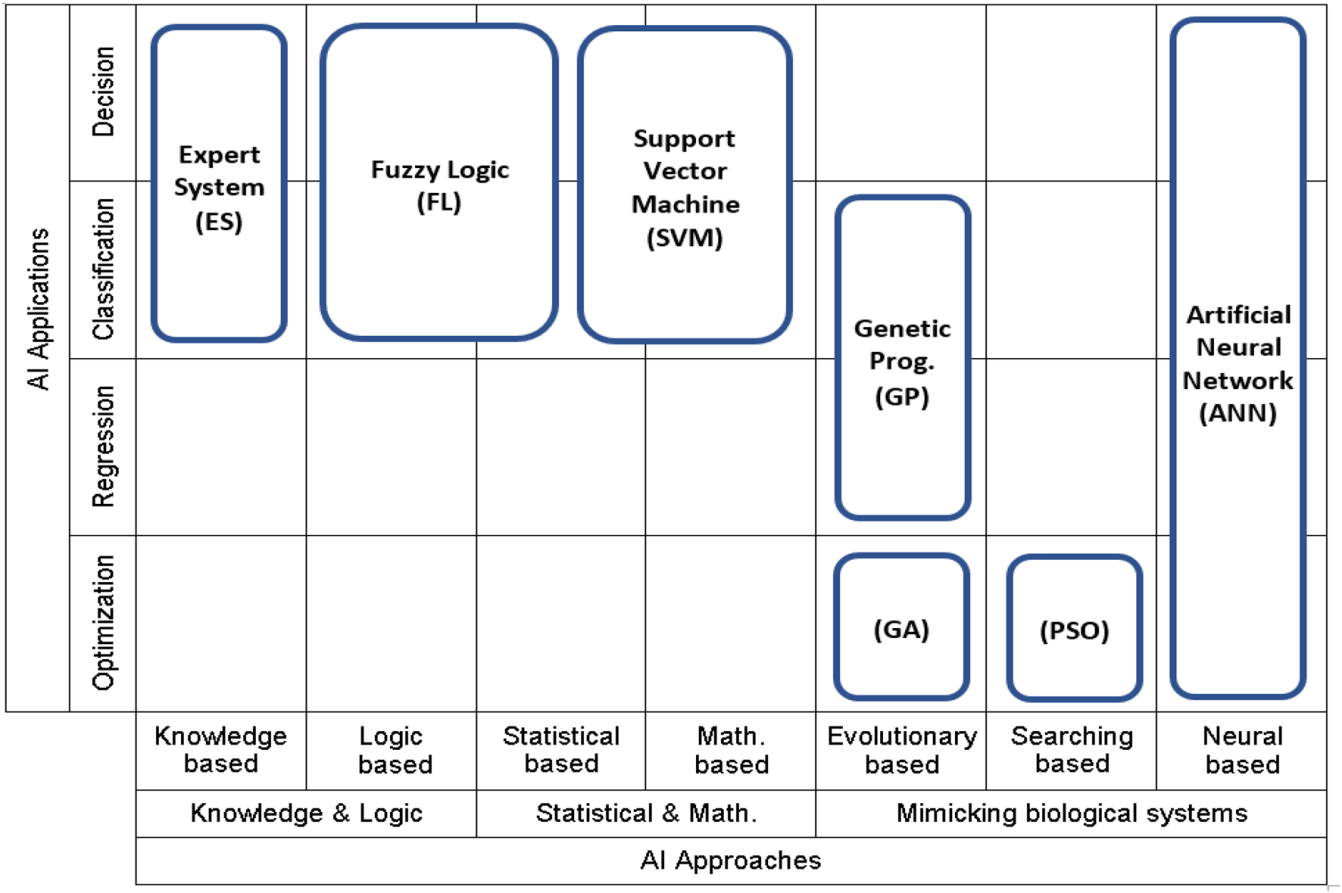
(Application-Approach) mapping for the considered (AI) techniques, After Ebid 2023.

AI techniques are the mathematical approaches developed to search for the optimal solution of complicated problems within the available time and resources (hardware, software and databases). The more time and resources allowed the more accurate and better solution AI can reach. There are two main types of AI approaches, one of them is based on mimicking the behavior of natural creatures and the other one depends on logical, mathematical or statistical approaches. Each type of them can deal better with specific types of problem. The first one (such as ANN, GA, GP and PSO) is better for inaccurate, distorted and incomplete data, while the other one (such as ES, FL and SVM) will be better for problems that need proof and reason. Accordingly, the suitable technique for certain problem could be selected based on problem type, data quality, data distribution and search restrictions. Figure 12 presents a classification for considering AI techniques by approaches and applications.

#### Artificial neural networks (ANN)

This is the most successful and famous AI techniques. It depends on simulating the human brain anatomy and operation. The network consists of sets of cells (called neurons) arranged in layers. The cells of each layer are connected to the cells of the previous and the next layers. In human brain, electrical signals transfer between cells through the connectors, accordingly, they are affected by the quality of the connectors. On the other hand, the cells don't trigger their output signals until the summation of the input signals exceeded a certain thresholds. Similarly, the inputs of ANN transfer between neurons through the connectors. The quality of the biological connector is simulated by the weight of the ANN connector, and to simulate the effect of connector quality on the electrical signal, the end ANN neuron receives the input value multiplied by the connector weight. Finally, the threshold of biological cell is simulated by the "activation function" in ANN neuron which trigger the neuron output. Just like the human brain, the ANN gains knowledge by learning, during this process, the ANN adjusts the weight of each connector to maximize the accuracy of the outputs.

#### Genetic programming (GP)

GP is a special application of GA where the considered problem is to optimize a mathematical expression to fit certain observations, hence GP is a multi-variable and free structure regression technique. The individual solutions of GA are presented by mathematical expressions in GP and the fitness function of GA is replaced by the Sum of Squared Errors (SSE) in GP. Finally, to apply GA on the mathematical expressions, they must be coded in genetic form first. Today, GP is a main technique includes many sub-techniques such as Linear and Cartesian Genetic Programming (LGP) & (CGP) besides Gene Expression Programming (GEP) and Multi- Gene Expression Programming (MGEP).

#### Evolutionary polynomial regression (EPR) 

EPR is an optimized polynomial regression using Genetic Algorithm (GA) technique. The transitional polynomial regression depends on calculating the best fitting polynomial coefficients mathematically, however, for high order polynomials with multi-variables applications the number of polynomial terms becomes hundreds and even thousands and the hence the results become unpractical. In EPR technique, GA is used to select the most influence polynomial terms from the total number of the terms which keeps the results accurate enough and applicable.

### Predicting the correction factors

Three different Artificial Intelligent (AI) techniques were used to predict the correction factors of both ACI-318 and EC2 punching capacity formulas (K Aci, K Ec2) of PT-UHPC slabs using the generated database. These techniques are “Genetic programming” (GP), “Artificial Neural Network” (ANN) and “Evolutionary Polynomial Regression” (EPR). All the three developed models were used to predict (K Aci, K Ec2) using “characteristic strength of concrete in MPa” (Fcu), “aspect ratio of column” (L/B), “tensile capacity of shear reinforcement in kN” (Ash.Fy) and “the average pre-stressing stress in both directions in kPa” (Fps).

Each model on the three developed models was based on a different approach (evolutionary approach for GP, mimicking biological neurons for ANN and optimized mathematical regression technique for EPR). These techniques were selected as they are the most suitable (AI) techniques for regression applications^32^. However, for all developed models, prediction accuracy was evaluated in terms of the Sum of Squared Errors (SSE).

The following section discusses the results of each model. The Accuracies of developed models were evaluated by comparing the (SSE) between predicted and calculated correction factors values. The results of all developed models are summarized in Table 10 and Fig. 15. While Fig. 16 presents a comparison between the accurizes of the developed models.

## Results 

### Using GP technique

The developed GP model has five levels of complexity. The population size, survivor size and number of generations were 100 000, 30 000 and 150 respectively. Equations 4 and 5 present the output formula for K Aci and K Ec2 respectively, while Fig. 15a,d showed their fitness. The average errors% of total dataset are 4.8% and 4.1%, while the R^2^ values are 0.943 and 0.965 in order.

4 $$ {\mathrm{KAci}} = 1 + \left( {\frac{{{\mathrm{Fcu}} \left( {{\mathrm{Fcu}} - {\mathrm{Aspect}}^{2} } \right)}}{21215}} \right)^{5} $$

5 $$ {\mathrm{KEc}}2 = \left( {\frac{1}{{116 - {\mathrm{Fcu}}}} + \frac{{{\mathrm{Fcu}} - 36}}{54}} \right)\left( {\frac{{10{\mathrm{Ash}}.{\mathrm{Fy}} - 10}}{{10{\mathrm{Ash}}.{\mathrm{Fy}} - 9}}} \right) - 0.07 $$

### Using ANN technique

**Figure 13:**
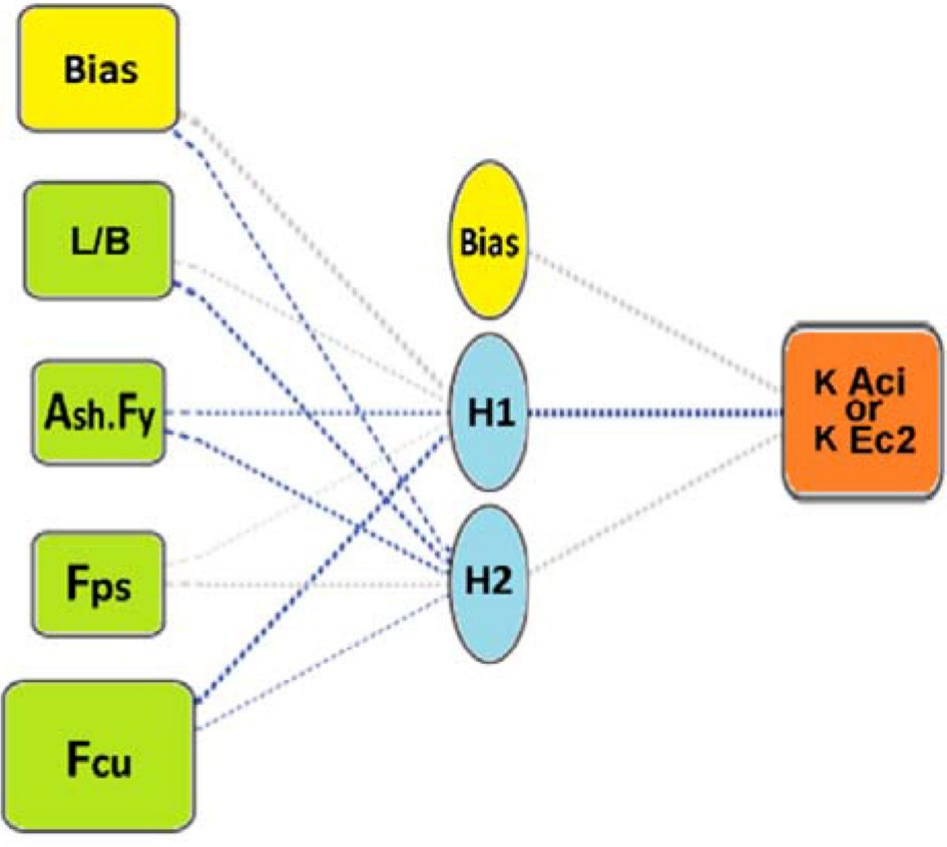
Layout for the developed ANN models.

**Figure 14:**
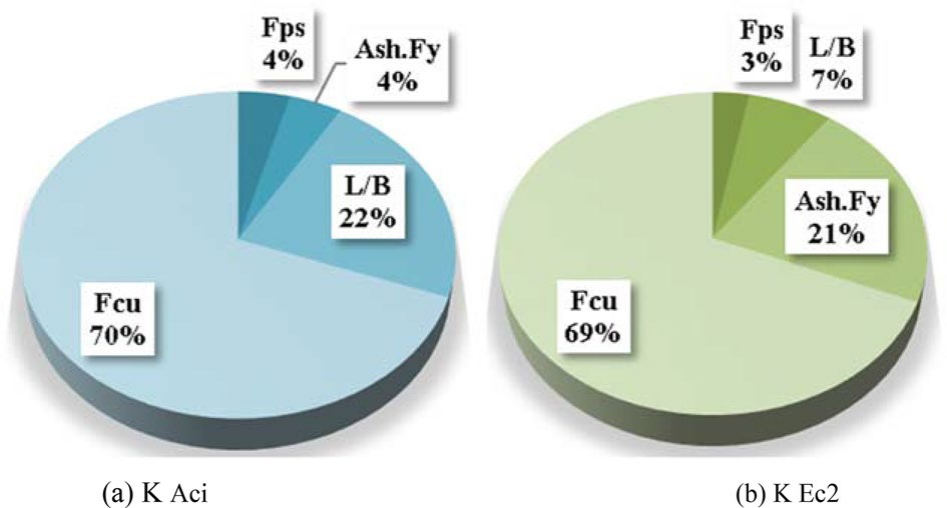
Relative importance of input parameters.

**Figure 15:**
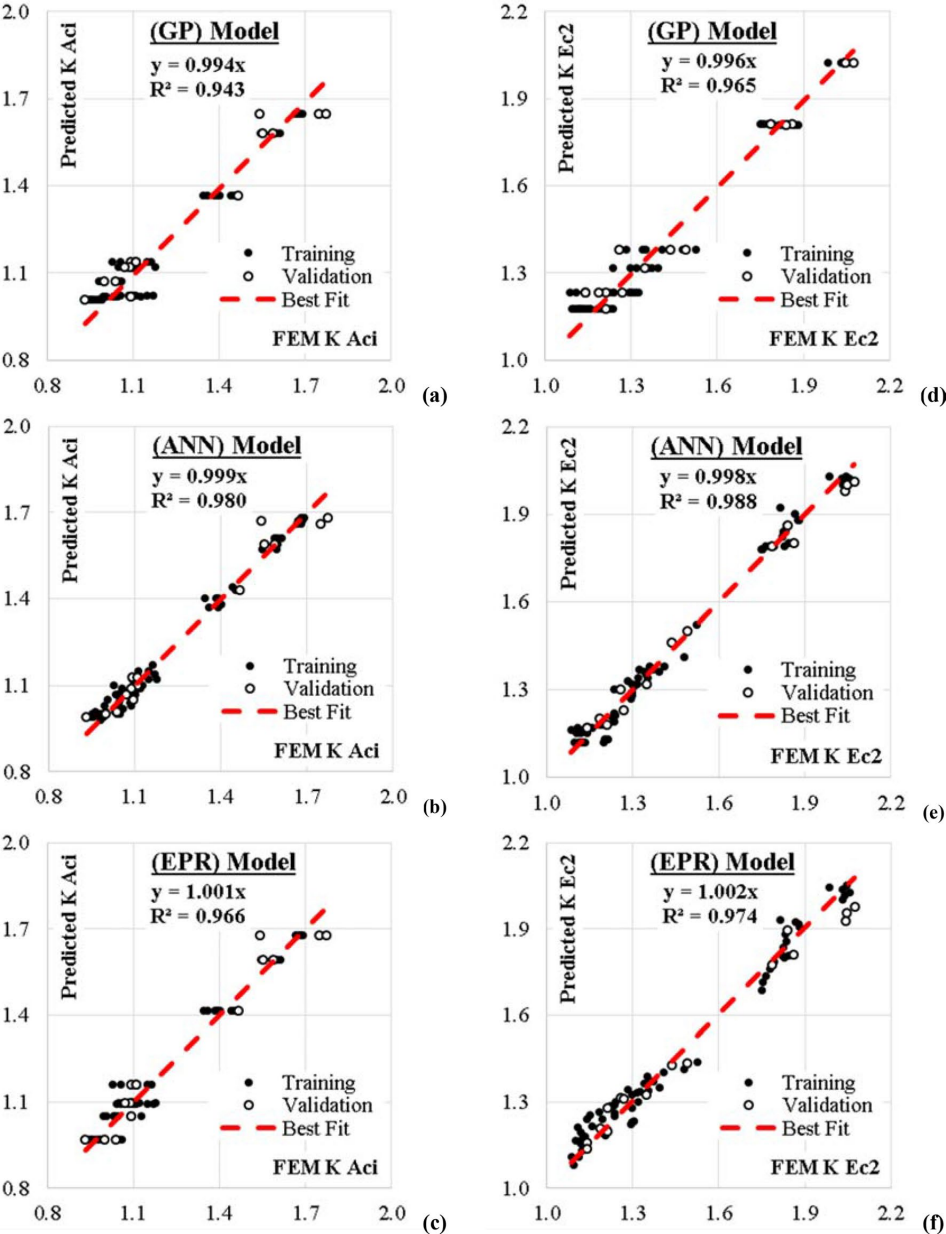
Relation between predicted and calculated (K Aci & K Ec2) values using the developed models.

Two models were developed using ANN technique, one to predict (K Aci) values and the other to predict (K Ec2) values. Both models used normalization method − 1.0 to 1.0, activation function Hyper Tan and “Back propagation” BP training algorithm. The used networks had the same layout as illustrated in Fig. 13 while the weight matrix of each model is shown in Tables 8 and 9. The average errors % of total dataset are 3.0%, 2.5% and the R^2^ values are 0.980, 0.988 respectively. The relative importance values for each input parameter are illustrated in Fig. 14, which indicated that both correction factors (K Aci & K Ec2) depended mainly on the “characteristic strength of the concrete” Fcu, In addition, “the aspect ratio” L/B showed significant impact on the (K Aci), while “ultimate tensile capacity of shear reinforcement” (Ash.Fy) significantly affected the (K Ec2). Finally, both “Aspect ratio of column” L/B and “average pre-stressing stress” Fps had neglected effect on both (K Aci & K Ec2). The relations between calculated and predicted values are shown in Fig. 15b,e.

**Table 8:** Weights matrix for the developed ANN For (K Aci).

	Bias	L/B	Ash.Fy	Fps	Fcu		K Aci
H (1)	1.61	0.12	− 0.06	0.02	− 1.38	H (1)	− 1.38
H (2)	− 0.33	− 0.55	− 0.20	0.19	− 0.05	H (2)	0.43
						Bias	0.58

**Table 9:** Weights matrix for the developed ANN For (K Ec2).

	Bias	L/B	Ash.Fy	Fps	Fcu		K Ec2
H (1)	− 0.81	2.74	0.24	0.26	0.03	H (1)	0.96
H (2)	− 0.54	− 0.16	0.18	− 1.02	0.13	H (2)	0.58
						Bias	0.04

### Using EPR technique.

Finally, the developed EPR model was limited to 5th level polynomial, for 4 inputs, there are 126 possible terms (70 + 35 + 15 + 5 + 1 = 126) as follows:

$$\sum_{m=1}^{m=4}\sum_{l=1}^{l=4}\sum_{k=1}^{k=4}\sum_{j=1}^{j=4}\sum_{i=1}^{i=4}{{{{X}_{m}.X}_{l}.X}_{k}.{X}_{j}.X}_{i}+\sum_{l=1}^{l=4}\sum_{k=1}^{k=4}\sum_{j=1}^{j=4}\sum_{i=1}^{i=4}{{{X}_{l}.X}_{k}.{X}_{j}.X}_{i}+\sum_{k=1}^{k=4}\sum_{j=1}^{j=4}\sum_{i=1}^{i=4}{{X}_{k}.{X}_{j}.X}_{i}+\sum_{j=1}^{j=4}\sum_{i=1}^{i=4}{X}_{j}.{X}_{i}+\sum_{i=1}^{i=4}{X}_{i}+C$$

GA technique was applied on these 126 terms to select the most effective five terms to predict the values of (K Aci & K Ec2) values. The output is illustrated in Eqs. 6 and 7 and the fitness of both models is shown in Fig. 15c,f. The average error % and R^2^ values were 3.9% & 3.5%—0.966 & 0.974 for (K Aci & K Ec2) respectively.

6 $$ {\mathrm{KAci}} = \left( {\frac{{{\mathrm{Fcu}}}}{12}} \right) + \left( {\frac{945}{{{\mathrm{Fcu}}}}} \right) - \left( {\frac{{{\mathrm{Fcu}}.{\mathrm{L}}}}{{850{\mathrm{B}}}}} \right) + \left( {\frac{{\mathrm{L}}}{{13{\mathrm{B}}}}} \right) - 16.65 $$

7 $$ {\mathrm{KEc}}2 = \left( {\frac{{{\mathrm{Fcu}}}}{10.1}} \right) + \left( {\frac{1150}{{{\mathrm{Fcu}}}}} \right){-} \left( {\frac{{{\mathrm{Fcu}}.{\mathrm{Ash}}.{\mathrm{Fy}}}}{63000}} \right){-} \left( {\frac{{40{\mathrm{B}}/{\mathrm{L}}}}{{{\mathrm{Fps}}}}} \right) - 20 $$

## Discussion

Longer spans required thicker and heavier slabs, to reduce the own weight, stronger concrete is used which lead to thinner slabs and smaller columns. This combination of thin slab and small columns has two disadvantages (1) large deflection and (2) punching problem. The deflection problem is solved using post-tension technology while the punching still a problem. The stronger the concrete (UHPC) the thinner the slab and the more serious punching problem, that is why this research focused on this point. Moreover, there is no design codes for (UHPC) nor (PT-UHPC), all the current design cods are limited to (Fc’ = 70 MPa) as mentioned in the introduction, accordingly, their predictions are not accurate, this research proposed correction factors to make the code formulas predictions more closer to the experimental results (more accurate).

During the conducted literature review surveying, many previous researches conferenced in UHPC slabs and PT slabs where found and summarized, however, no previous researches were fund regarding the punching capacity of PT-UHPC slabs except the two references published by the authors^2,3^. Those references were concerned in figure out the impact of four parameters on the punching capacity of the PT-UHPC slabs namely concrete strength (Fcu), shear reinforcement (Ash.Fy), average pre-stressing stress (Fps) and the aspect ratio of the column (L/B). The impact of each one of these parameters was discussed in details in terms of ultimate capacity, failure mechanism, stiffness and energy dissipation. Accordingly, these items will not be discussed again in this research, this discussion will be concerned in the outcomes of each phase of the used methodology could be discussed as follows:

The outcome of phase1 is the verified typical FEM model. The verifications results showed in Table 5 indicated that the FEM punching capacity equals the experimental one with maximum error ± 4% and ± 13% for deformations. These variations are very acceptable considering the random errors in experimental work due to measurements tolerances, variation in materials, temperature effects and many other minor factors. Accordingly, the FEM is considered verified. Comparing these variance with the design code formulas variances (± 60% for ACI & ± 100% for EC2) showed how accurate is the FEM.

By the end of phase 2, a complete parametric study were successfully generated as early planned before conducting the experimental work. The role of thumb is (3–10) reorders for each variables, accordingly the 81 records are quite enough for 4 variables. The most effective factors on punching and their ranges were determined from literature, the three values for each factor were chosen at lower, mid and upper boundaries of the range. About 10% of the whole parametric study (9 samples) was tested experimentally and the results were published in 2 papers and used to verify the FEM model, the rest 90% of the parametric study were tested numerically using the verified FEM.

The generated database was divided in to training set (80%) and validation set (20%) as recommended in literatures. then both sets were statistically analyzed to insure that they have the almost the same statistical characteristics and that the selected parameters values covers all the considered range without gaps. Besides that, Pearson correlation analysis indicated the impact ranking of each parameter.

**Figure 16:**
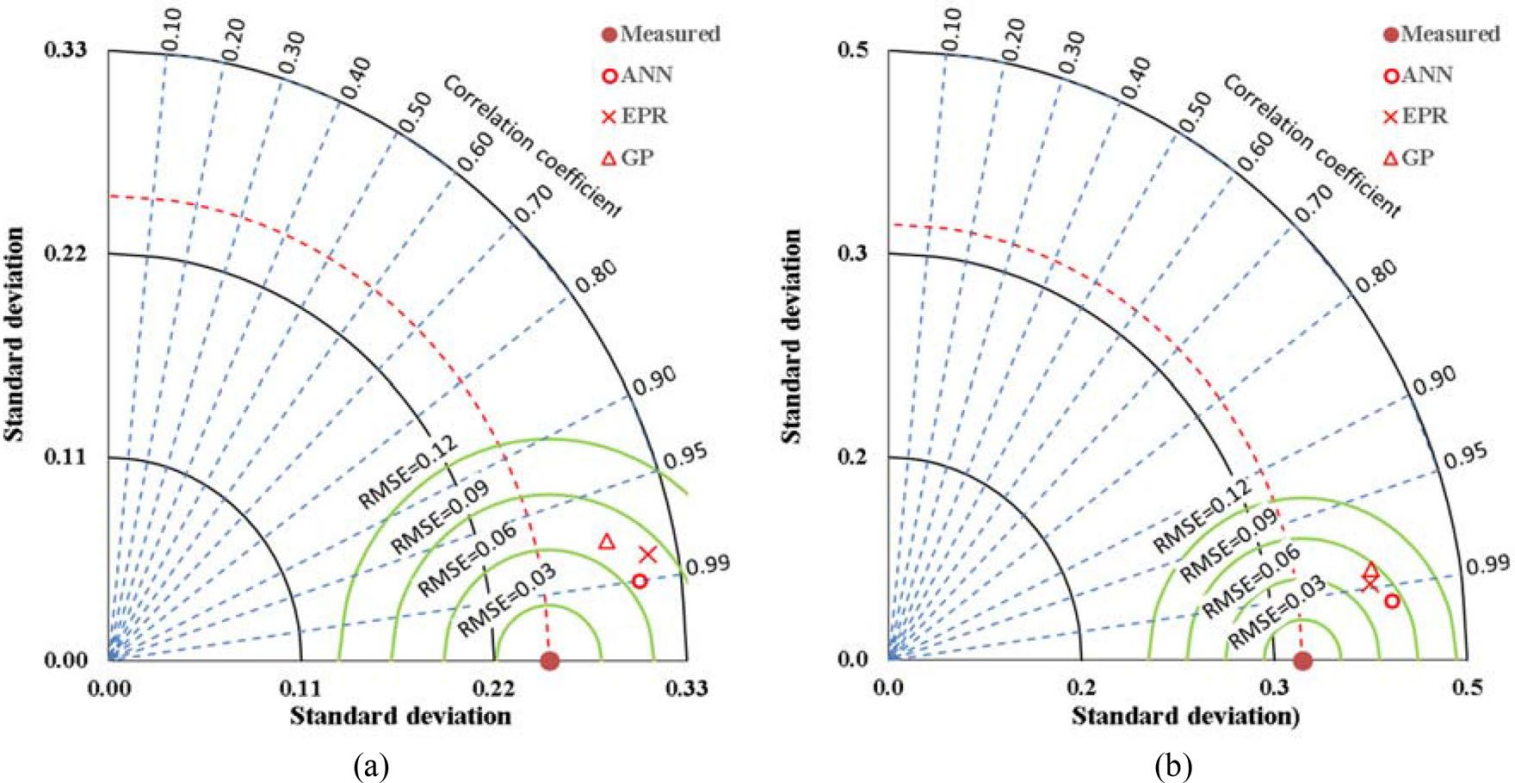
Comparing the accuracies of the developed models using Taylor charts.

Finally, the outcomes of phase 3 are three predictive models for the correction factors of ACI-318 and EN2 codes. The utilized techniques were selected as they are the most suitable (AI) techniques for regression applications and because they depends on different (AI) approaches. The fittings of the three developed models are graphically presented in Fig. 15, summarized in Table 10 and compared using Tylor charts in Fig. 16.

**Table 10:** Accuracies of developed models.

Item	Technique	Model	SSE	Avg. Error %	R^2^
K Aci	GP	Equation 4	0.259	4.8	0.943
	ANN	Figure 9(a), Table 6	0.100	3.0	0.980
	EPR	Equation 6	0.174	3.9	0.966
K Ec2	GP	Equation 5	0.269	4.1	0.965
	ANN	Figure 9(b), Table 7	0.102	2.5	0.988
	EPR	Equation 7	0.200	3.5	0.974

The 16 records validation dataset (20% of the total dataset) was used to test the fitting of all the predictive models and to insure that there is no over-fitting. The results of both training dataset and validation dataset were graphically presented side by side in the same chart (black and white dots) in Fig. 15 to illustrate that both sets had the same fitting. These charts showed that the best fitting line id almost at 45 degrees (y ≈ x) which means that the mean values from FEM and predictive models are the same, Also, the (R^2^) values are more than 0.9 which indicated a limited scattering from the mean values.

Table 10 compares the fittings of the predictive models in terms of (SSE) and (Error %) where (Error% = RMSE/Mean), accordingly the prediction accuracy may be defined as (1-Error%). The numbers indicated that ANN is the most accurate model then the EPR and lastly the GP.

Tylor charts in Fig. 16 compared the fitting of the predicting models based on three statistical measurements RSME, SD and correlation coefficient. The closer the prediction point to the experimental one the better performance of the technique, besides that, the higher the correlation coefficient the better the model fitting. Accordingly, the charts indicated the same order as Table 10. However, the fitting is not the only strength point for the predictive model, simplicity and applicability are also respectable strength points. From simplicity point of view, GP & EPR are much simpler that ANN and more manually applicable since they are both closed form equations.

## Conclusions

This paper proposes correction factors for ACI-318 and EC2 design codes to extend the validity of their punching capacity provisions to include the post tensioned ultrahigh performance concrete PT-UHPC flat slabs. These correction factors were developed using three different Machine Learning (ML) techniques. The utilised database in the ML models was generated using FEM parametric study which used a validated FEM model against experimental test results from previously published work. The proposed correction factors are functions of the concrete compressive strength (Fcu), PT strands layout (in terms of the average pre-stressing stress in the punching zone) (Fps), shear reinforcement capacity (Ash.Fy) and the aspect ratio of the column (L/B). The outcomes of this research could be concluded as follows:

- The developed finite element model is successfully validated against the experimentally tested PT-UHPC flat slabs in terms of ultimate punching capacity and midpoint deflection with error of 3.8% and 12.7% respectively.
- All models showed almost the same level of accuracy (95–97%) for the ACI-318 correction factor (K Aci) and (96–97.5%) for the EC2 correction factor (K Ec2)
- Using the proposed PT-UHPC correction factors with original codes formulas for punching capacity enhances the prediction accuracy from about (50%- 60%) to more than (95%)
- Although the predicting accuracies are almost the same for all models, but (GP) & (EPR) models have the advantage of simplicity as their outputs are closed form formula which could be used manually unlike the outputs of the (ANN) mode which are two weight matrixes that can’t be implemented manually.
- The results indicated that both correction factors (K Aci & K Ec2) depended mainly on the “characteristic strength of the concrete” (Fcu). In addition, “the aspect ratio” (L/B) showed significant impact on the (K Aci), while “the tensile capacity of shear reinforcement” (Ash.Fy) significantly affected the (K Ec2). Finally, “the average pre-stressing stress” (Fps) had neglected effect on both (K Aci & K Ec2).
- Like any other regression technique, the generated formulas are valid within the considered range of parameter values, beyond this range; the prediction accuracy should be verified.

## Possible directions for future studies

- Extend the validation of the developed correction factors as this research is limited to concrete with compressive strength of 100 MPa, 120 MPa and 140 MPa.
- Investigate of the influence of other parameters such as different levels of prestressing.
- Applying UHPC with mild reinforcement only (no prestressing)
- Modification of the current applied design codes equations.

## Supplementary Information

Supplementary Information.
