# Supplementary material for: Machine learning base models to predict the punching shear capacity of posttensioned UHPC flat slabs
Source: Sci Rep. 2024 Feb 17;14:3969. doi: 10.1038/s41598-024-54358-5 (PMC10874378; doi:10.1038/s41598-024-54358-5)
Supplement: Supplementary file 1 — Supplementary Information. [file 41598_2024_54358_MOESM1_ESM.docx]

**Appendix (Utilized database)**

| **Fcu** | **L/B** | **Ash.Fy** | **Fps** | **K Aci** | **K Ec2** |
| --- | --- | --- | --- | --- | --- |
| **MPa** | **(-)** | **(kN)** | **(kPa)** | **(-)** | **(-)** |
| **Training set** | | | | | |
| 120.00 | 4.00 | 54.00 | 267.00 | 0.98 | 1.30 |
| 120.00 | 1.00 | 0.00 | 400.00 | 1.16 | 1.34 |
| 140.00 | 4.00 | 108.00 | 267.00 | 1.44 | 1.83 |
| 100.00 | 4.00 | 0.00 | 333.00 | 0.97 | 1.37 |
| 100.00 | 1.00 | 108.00 | 400.00 | 1.11 | 1.12 |
| 120.00 | 4.00 | 54.00 | 400.00 | 1.00 | 1.32 |
| 100.00 | 1.00 | 108.00 | 333.00 | 1.10 | 1.11 |
| 100.00 | 2.00 | 108.00 | 267.00 | 1.03 | 1.12 |
| 140.00 | 1.00 | 108.00 | 333.00 | 1.68 | 1.75 |
| 140.00 | 4.00 | 54.00 | 400.00 | 1.35 | 1.82 |
| 120.00 | 2.00 | 54.00 | 333.00 | 1.06 | 1.24 |
| 100.00 | 2.00 | 54.00 | 267.00 | 0.99 | 1.14 |
| 140.00 | 2.00 | 0.00 | 267.00 | 1.59 | 2.03 |
| 100.00 | 1.00 | 0.00 | 267.00 | 1.10 | 1.24 |
| 100.00 | 4.00 | 108.00 | 267.00 | 0.98 | 1.20 |
| 120.00 | 2.00 | 0.00 | 400.00 | 1.18 | 1.48 |
| 120.00 | 4.00 | 108.00 | 333.00 | 1.04 | 1.30 |
| 100.00 | 4.00 | 0.00 | 400.00 | 0.96 | 1.36 |
| 140.00 | 1.00 | 54.00 | 333.00 | 1.67 | 1.83 |
| 120.00 | 4.00 | 0.00 | 400.00 | 1.06 | 1.52 |
| 120.00 | 1.00 | 54.00 | 400.00 | 1.15 | 1.24 |
| 140.00 | 1.00 | 108.00 | 267.00 | 1.68 | 1.75 |
| 100.00 | 4.00 | 54.00 | 400.00 | 0.95 | 1.24 |
| 100.00 | 1.00 | 0.00 | 400.00 | 1.17 | 1.32 |
| 140.00 | 2.00 | 108.00 | 400.00 | 1.60 | 1.79 |
| 120.00 | 1.00 | 54.00 | 267.00 | 1.03 | 1.11 |
| 140.00 | 2.00 | 54.00 | 267.00 | 1.54 | 1.83 |
| 100.00 | 2.00 | 108.00 | 400.00 | 1.05 | 1.13 |
| 100.00 | 4.00 | 108.00 | 400.00 | 0.99 | 1.21 |
| 140.00 | 1.00 | 54.00 | 400.00 | 1.68 | 1.84 |
| 100.00 | 2.00 | 0.00 | 400.00 | 1.13 | 1.39 |
| 120.00 | 2.00 | 0.00 | 333.00 | 1.12 | 1.41 |
| 140.00 | 2.00 | 54.00 | 400.00 | 1.59 | 1.88 |
| 100.00 | 1.00 | 54.00 | 333.00 | 1.06 | 1.12 |
| 140.00 | 2.00 | 0.00 | 333.00 | 1.60 | 2.04 |
| 120.00 | 4.00 | 54.00 | 333.00 | 0.99 | 1.32 |
| 120.00 | 1.00 | 0.00 | 333.00 | 1.11 | 1.28 |
| 120.00 | 4.00 | 108.00 | 267.00 | 1.04 | 1.29 |
| 140.00 | 1.00 | 54.00 | 267.00 | 1.66 | 1.82 |
| 140.00 | 1.00 | 108.00 | 400.00 | 1.69 | 1.76 |
| 140.00 | 4.00 | 0.00 | 333.00 | 1.36 | 1.99 |
| 140.00 | 4.00 | 108.00 | 333.00 | 1.45 | 1.84 |
| 100.00 | 2.00 | 54.00 | 333.00 | 1.01 | 1.15 |
| 100.00 | 2.00 | 108.00 | 333.00 | 1.04 | 1.12 |
| 100.00 | 1.00 | 108.00 | 267.00 | 1.09 | 1.10 |
| 100.00 | 4.00 | 108.00 | 333.00 | 0.99 | 1.20 |
| 140.00 | 2.00 | 0.00 | 400.00 | 1.61 | 2.06 |
| 120.00 | 2.00 | 108.00 | 267.00 | 1.09 | 1.20 |
| 120.00 | 4.00 | 108.00 | 400.00 | 1.05 | 1.31 |
| 100.00 | 4.00 | 54.00 | 333.00 | 0.95 | 1.23 |
| 120.00 | 2.00 | 54.00 | 267.00 | 1.05 | 1.22 |
| 100.00 | 1.00 | 54.00 | 400.00 | 1.09 | 1.16 |
| 140.00 | 4.00 | 0.00 | 267.00 | 1.39 | 2.03 |
| 140.00 | 4.00 | 0.00 | 400.00 | 1.40 | 2.04 |
| 100.00 | 2.00 | 0.00 | 333.00 | 1.10 | 1.35 |
| 140.00 | 4.00 | 54.00 | 267.00 | 1.39 | 1.88 |
| 100.00 | 4.00 | 0.00 | 267.00 | 0.96 | 1.35 |
| 120.00 | 2.00 | 0.00 | 267.00 | 1.07 | 1.35 |
| 100.00 | 1.00 | 0.00 | 333.00 | 1.15 | 1.30 |
| 120.00 | 1.00 | 54.00 | 333.00 | 1.11 | 1.19 |
| 100.00 | 1.00 | 54.00 | 267.00 | 1.04 | 1.10 |
| 100.00 | 2.00 | 54.00 | 400.00 | 1.03 | 1.18 |
| 140.00 | 2.00 | 108.00 | 267.00 | 1.58 | 1.78 |
| 140.00 | 4.00 | 54.00 | 333.00 | 1.38 | 1.87 |
| 120.00 | 1.00 | 108.00 | 267.00 | 1.06 | 1.09 |
| **Validation set** | | | | | |
| 100.00 | 2.00 | 0.00 | 267.00 | 1.09 | 1.35 |
| 140.00 | 4.00 | 108.00 | 400.00 | 1.47 | 1.86 |
| 120.00 | 1.00 | 0.00 | 267.00 | 1.09 | 1.26 |
| 120.00 | 1.00 | 108.00 | 400.00 | 1.11 | 1.14 |
| 120.00 | 2.00 | 108.00 | 333.00 | 1.10 | 1.21 |
| 120.00 | 1.00 | 108.00 | 333.00 | 1.11 | 1.14 |
| 140.00 | 2.00 | 54.00 | 333.00 | 1.55 | 1.84 |
| 120.00 | 4.00 | 0.00 | 267.00 | 1.00 | 1.44 |
| 140.00 | 1.00 | 0.00 | 267.00 | 1.75 | 2.04 |
| 100.00 | 4.00 | 54.00 | 267.00 | 0.93 | 1.21 |
| 140.00 | 1.00 | 0.00 | 333.00 | 1.54 | 2.05 |
| 120.00 | 4.00 | 0.00 | 333.00 | 1.04 | 1.49 |
| 140.00 | 2.00 | 108.00 | 333.00 | 1.59 | 1.78 |
| 120.00 | 2.00 | 54.00 | 400.00 | 1.09 | 1.27 |
| 140.00 | 1.00 | 0.00 | 400.00 | 1.77 | 2.07 |
| 120.00 | 2.00 | 108.00 | 400.00 | 1.07 | 1.19 |
